# A Comprehensive Analysis of SE-lncRNA/mRNA Differential Expression Profiles During Chondrogenic Differentiation of Human Bone Marrow Mesenchymal Stem Cells

**DOI:** 10.3389/fcell.2021.721205

**Published:** 2021-09-13

**Authors:** Yu Jiang, Chen Zhang, Lujue Long, Lihua Ge, Jing Guo, Zhipeng Fan, Guoxia Yu

**Affiliations:** ^1^Department of Stomatology, Beijing Children’s Hospital, Capital Medical University, National Center for Children’s Health, Beijing, China; ^2^Laboratory of Molecular Signaling and Stem Cells Therapy, Beijing Key Laboratory of Tooth Regeneration and Function Reconstruction, School of Stomatology, Capital Medical University, Beijing, China; ^3^Hunan Key Laboratory of Oral Health Research, Hunan 3D Printing Engineering Research Center of Oral Care, Hunan Clinical Research Center of Oral Major Diseases and Oral Health, Xiangya Stomatological Hospital, Xiangya School of Stomatology, Central South University, Hunan, China; ^4^The Key Laboratory of Oral Biomedicine, The Affiliated Stomatological Hospital of Nanchang University, Nanchang, China; ^5^Research Unit of Tooth Development and Regeneration, Chinese Academy of Medical Sciences, Beijing, China

**Keywords:** MSC, gene expression, super enhancer long non-coding RNA, mRNA, chondrogenic differentiation

## Abstract

**Objective:** Articular cartilage injury is common and difficult to treat clinically because of the characteristics of the cartilage. Bone marrow-derived mesenchymal stem cell (BMSC)-mediated cartilage regeneration is a promising therapy for treating articular cartilage injury. BMSC differentiation is controlled by numerous molecules and signaling pathways in the microenvironment at both the transcriptional and post-transcriptional levels. However, the possible function of super enhancer long non-coding RNAs (SE-lncRNAs) in the chondrogenic differentiation of BMSCs is still unclear. Our intention was to explore the expression profile of SE-lncRNAs and potential target genes regulated by SE-lncRNAs during chondrogenic differentiation in BMSCs.

**Materials and Methods:** In this study, we conducted a human Super-Enhancer LncRNA Microarray to investigate the differential expression profile of SE-lncRNAs and mRNAs during chondrogenic differentiation of BMSCs. Subsequent bioinformatic analysis was performed to clarify the important signaling pathways, SE-lncRNAs, and mRNAs associated with SE-lncRNAs regulating the chondrogenic differentiation of BMSCs.

**Results:** A total of 77 SE-lncRNAs were identified, of which 47 were upregulated and 30 were downregulated during chondrogenic differentiation. A total of 308 mRNAs were identified, of which 245 were upregulated and 63 were downregulated. Some pathways, such as focal adhesion, extracellular matrix (ECM)–receptor interaction, transforming growth factor-β (TGF-β) signaling pathway, and PI3K–Akt signaling pathway, were identified as the key pathways that may be implicated in the chondrogenic differentiation of BMSCs. Moreover, five potentially core regulatory mRNAs (PMEPA1, ENC1, TES, CDK6, and ADIRF) and 37 SE-lncRNAs in chondrogenic differentiation were identified by bioinformatic analysis.

**Conclusion:** We assessed the differential expression levels of SE-lncRNAs and mRNAs, along with the chondrogenic differentiation of BMSCs. By analyzing the interactions and co-expression, we identified the core SE-lncRNAs and mRNAs acting as regulators of the chondrogenic differentiation potential of BMSCs. Our study also provided novel insights into the mechanism of BMSC chondrogenic and cartilage regeneration.

## Introduction

Articular cartilage injury is a common and serious condition accompanied by degeneration or loss of cartilage, which can cause swelling, pain, and even disability (Yu et al., 2018). Many reasons, including trauma, tumors, and osteoarthritis, can cause articular cartilage damage. Articular cartilage, a connective tissue, is composed of chondrocytes and cartilage extracellular matrix (ECM) (Krishnan and Grodzinsky, 2018). Thus, self-repair is difficult for damaged cartilage because of its avascular, aneural, and alymphatic properties and the low mitotic ability of chondrocytes (Carballo et al., 2017; Yu et al., 2018; Tan et al., 2021). Articular cartilage injury presents a huge therapeutic challenge. In recent years, advances in stem cell therapy have offered possibilities for cartilage repair (Zhang et al., 2014; Tan et al., 2021). At present, it has been shown that bone marrow-derived mesenchymal stem cell (BMSC) therapy is safe and effective for the treatment of cartilage damage (Wong et al., 2013; Teo et al., 2019). Owing to its strong self-renewal, multidirectional differentiation potential, and immunomodulatory properties, BMSCs are regarded as promising seed cells for stem cell therapy (Zhang et al., 2017). BMSCs exhibit the potential to differentiate into multiple cell types, including chondrocytes, osteoblasts, and adipocytes, under different induction conditions (Cai et al., 2017). However, the mechanism of BMSC-targeted differentiation remains a complex event to be explored.

Along with technological developments, super enhancers (SEs), which are associated with pluripotency genes, were described for the first time in mouse embryonic stem cells in 2013 (Hnisz et al., 2013; Whyte et al., 2013). SEs are defined as a cluster of transcriptional enhancers that can span more than 20 kb (Tang F. et al., 2020). In contrast to typical enhancers that function independently, SEs can recruit a large number of transcription factors, mediators, and other proteins and greatly promote the transcription of target genes by multiple enhancers acting together (Hnisz et al., 2013; Pott and Lieb, 2015). Genes that play a determinant role in cell identification can be driven by SEs, which have a stronger ability for transcriptional activation than typical enhancers (Rhie et al., 2016; Jia et al., 2019; Wu and Shen, 2019). Moreover, SEs can also affect cell development and differentiation (Siersbæk et al., 2014; Pott and Lieb, 2015; Kitagawa et al., 2017). The expression of key oncogenes and other disease-related genes can also be driven by SEs (Willi et al., 2017; Pantera et al., 2018; Zhang C. et al., 2020). In addition to regulating the expression of target genes, SEs can actively transcribe and generate non-coding RNAs called SE RNAs, including lncRNAs, circRNAs, and miRNAs (Tan et al., 2020).

It has been found by many studies that SE-lncRNAs participate in the regulation of varieties of biological processes and many diseases by regulating target genes, including muscle, cardiac and red blood cell development, or cancer and myocardial infarction. The expression of Hand2, an important regulator of differentiation from fibroblasts to cardiomyocytes, is controlled by upstream SE-lncRNA Uph (Anderson et al., 2016). SE-lncRNA CCAT1-L interacts with CTCF and mediate long-range promoter–enhancer interactions at MYC loop regions to regulate MYC expression in colorectal cancer (Xiang et al., 2014; Zhang X. et al., 2020). SE-lncRNA UCA1-AMOT interacts with and activates the target gene YAP to promote ovarian cancer development (Lin et al., 2019). Given their prevalence and powerful functions, SE-lncRNAs have become one of the current research hotspots. However, studies on SE-lncRNAs in cell development and differentiation are still in their infancy. Currently, there are no studies on the effects of SE-lncRNAs and mRNAs associated with SE-lncRNAs during chondrogenic differentiation of BMSCs.

In this study, we aimed to explore the expression profile of SE-lncRNAs and potential target genes regulated by SE-lncRNAs during chondrogenic differentiation of BMSCs. Thus, the expression profiles of differentially expressed SE-lncRNAs and mRNAs were identified between uninduced and chondrogenic differentiation of human BMSCs for 7 days by performing microarray analysis. We further conducted bioinformatic analysis to analyze SE-lncRNAs–mRNAs interactions and identified potential core regulatory factors.

## Materials and Methods

### BMSC Culture and Chondrogenic Differentiation

Bone marrow-derived mesenchymal stem cells (three donors) were purchased from ScienCell Research Laboratories Inc. (Carlsbad, CA, United States). The culture medium was mesenchymal stem cell medium (MSCM) (ScienCell, Carlsbad, CA, United States) supplemented with 5% fetal bovine serum and 1% penicillin/streptomycin. The culture conditions were as described (Zhang et al., 2017). At three to five passages, BMSCs were used for the next detection. BMSCs were categorized into two groups: a group of uninduced BMSCs (*n* = 3) and a group of chondrogenic induction BMSCs (*n* = 3). Each pair of the uninduced and chondrogenic induction BMSCs were from the same donors. The uninduced group was cultured using MSCM. Chondrogenic induction BMSCs were cultured with a StemPro chondrogenesis differentiation kit (Invitrogen, Carlsbad, CA, United States) for 7 days. The induction medium was renewed every 3 days.

### RNA Isolation and Microarray Analysis

Total RNA from three samples from the two groups was extracted using the TRIzol reagent (Invitrogen). NanoDrop ND-1000 was used to determine RNA quantity and quality. Standard denaturing agarose gel electrophoresis was used to assess RNA integrity. Arraystar Human Super-Enhancer LncRNA Microarray including 7,753 lncRNAs and 7,040 genes was selected to acquire a comprehensive global profile of human lncRNAs and mRNAs. Recognized public transcriptome databases, such as RefSeq, UCSC knowledge genes, Ensemble, and important publications were used to build the lncRNA data. SE data were arranged from the database of dbSUPER. Samples were labeled and arrays were hybridized according to the Agilent One-Color Microarray-Based Gene Expression Analysis protocol (Agilent Technology). The Agilent DNA Microarray Scanner (part number G2505C) was used to scan after the array was washed and fixed. The acquired images were analyzed using the Agilent Feature Extraction software (version 11.0.1.1). The GeneSpring GX v12.1 software package (Agilent Technologies) was used for quantile normalization of raw data. Next, we selected eligible SE-lncRNAs and mRNAs for further analysis.

### Real-Time RT-PCR

The levels of genes that were differentially expressed in the microarray analysis were validated by real-time polymerase chain reaction (RT-PCR). Total RNA from the two groups was extracted as described above. RNA (1 μg) was combined with reverse transcriptase to synthesize cDNA according to the protocol of the manufacturer. QuantiTect SYBR Green PCR kits (Qiagen, Hilden, Germany) and an iCycler iQ multicolor real-time PCR detection system (Bio-Rad, Hercules, CA, United States) were used to perform real-time RT-PCR. The primers used are listed in Supplementary Table 1. mRNA expression was evaluated using the 2^–ΔΔCT^ method. The PCR signals were normalized by GAPDH.

### Bioinformatic Analysis

Fold change ≥ 2.0 and *p* ≤ 0.05 were considered as differentially expressed SE-lncRNAs and mRNAs between uninduced and chondrogenic induction BMSCs. We performed pathway and gene ontology (GO) analyses to determine the functions of important mRNAs in pathways or GO terms involved in chondrogenic differentiation. In-house scripts were used for hierarchical clustering and combined analyses. We built the pathway network (Path-Net) of the significant pathways associated with differentially expressed genes, which can reflect the association among these pathways according to the KEGG database. Based on the differentially expressed genes, the gene signal transduction network (signal-net) was constructed to determine the relationships between genes. To build a coding-non-coding co-expression (CNC) network, we calculated the correlation coefficient based on the normalized signal value between each pair of mRNAs and SE-lncRNAs.

### Statistics

Data were analyzed by Student’s *t*-test using SPSS17 statistical software (SPSS Inc., Chicago, IL, United States). Statistical significance was set at *p* ≤ 0.05.

## Results

### The Expression Profiles of Differential SE-lncRNAs and mRNAs During Chondrogenic Differentiation of BMSCs

**FIGURE 1:**
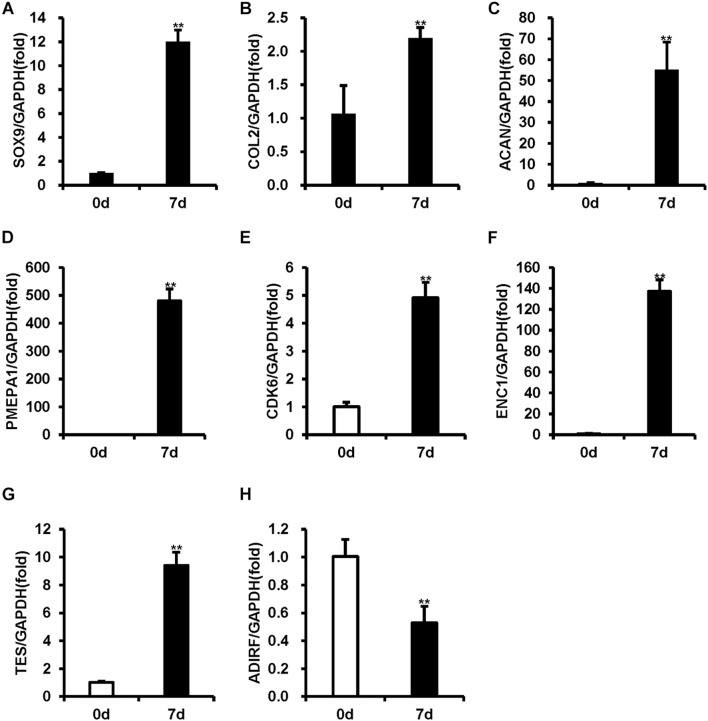
The mRNA levels were measured using real-time RT-PCR in BMSCs during chondrogenic differentiation. **(A–C)** The expression of SOX9, COL2, and ACAN was upregulated after 7 days of chondrogenic induction. **(D–G)** PMEP1, CDK6, ENC1, and TES expression increased after 7 days of chondrogenic induction. **(H)** ADIRF expression decreased after 7 days of chondrogenic induction. GAPDH acted as an internal reference. Statistical significance was determined by Student’s *t*-test. SD is represented by bars. (*n* = 3). **p < 0.01.

To verify the chondrogenic differentiation of BMSCs, real-time RT-PCR was used to measure the expression levels of the chondrogenic differentiation markers (SOX9, COL2, and ACAN). As shown in Figures 1A–C, 7-day chondrogenic induction BMSCs expressed higher levels of SOX9, COL2, and ACAN compared uninduced BMSCs (Figures 1A–C).

Student’s *t*-test was used to present the differentially expressed SE-lncRNAs and mRNAs. Based on the *p*-value threshold false discovery rate (FDR), we filtered out SE-lncRNAs and mRNAs that changed at least two-fold during chondrogenic differentiation of BMSCs for further analysis. In total, we found 77 differentially expressed SE-lncRNAs (*p* < 0.05, FDR < 0.05) between 7-day chondrogenic induction BMSCs and uninduced BMSCs of which 47 were upregulated and 30 were downregulated (Supplementary Table 2). Moreover, we identified 308 differentially expressed mRNAs (*p* < 0.05, FDR < 0.05) when comparing 7-day chondrogenic induction BMSCs with uninduced BMSCs. There were 245 upregulated genes, including EGR1, RUNX1, and ADAM12, and 63 downregulated genes, including SFRP1, IGFBP6, and ADRB2 (Supplementary Table 2).

To validate the accuracy of the microarray results, five mRNAs with the most obvious differential expression (PMEPA1, CDK6, ENC1, TES, and ADIRF) were selected, and real-time RT-PCR was used to measure their expression levels. Compared to the uninduced BMSCs, the induced BMSCs displayed increased expression levels of PMEPA1, CDK6, ENC1, and TES and decreased expression levels of ADIRF (Figures 1D–H). The expression levels of PMEPA1, CDK6, ENC1, TES, and ADIRF obtained by RT-PCR were in concordance with our microarray results.

### Bioinformatic Analysis of Microarray Data During Chondrogenic Differentiation of BMSCs

**FIGURE 2:**
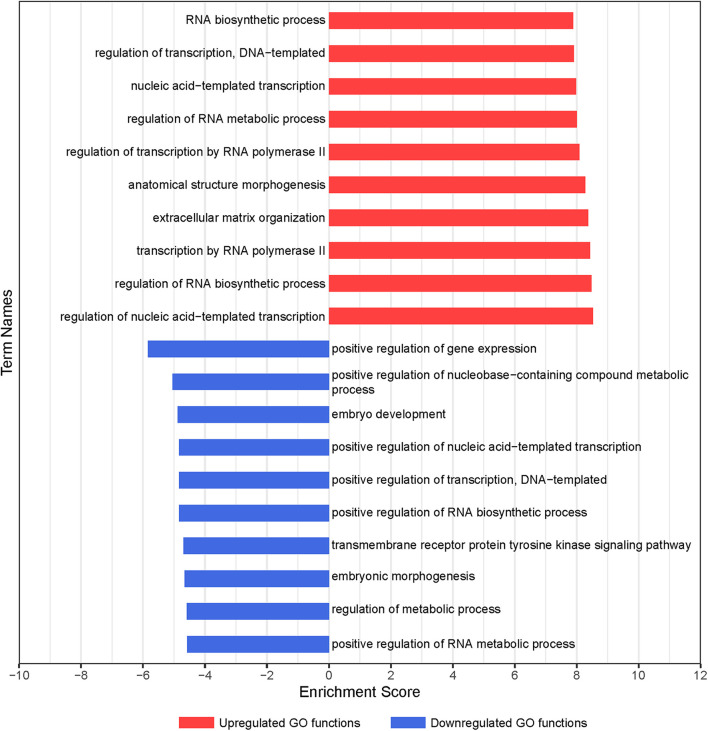
GO analysis of differentially expressed genes during chondrogenic differentiation of BMSCs. Significant GO biological process terms are represented on the *y*-axis. –LgP, negative logarithm of the *p*-value, are represented on the *x*-axis. Larger –LgP displays a smaller *p*-value.

To clarify the critical regulators governing chondrogenic differentiation of BMSCs, GO and pathway analyses were conducted. The significantly altered functions of differentially expressed genes during chondrogenic differentiation of BMSCs were identified by GO analysis. In total, 217 differential GO functions (*p* < 0.05, FDR < 0.05) were identified between 7-day chondrogenic induction BMSCs and uninduced BMSCs, of which 178 were upregulated and 39 were downregulated (Supplementary Table 3). –LgP, the negative logarithm of the *p*-value, was applied to show the correlation between gene expression and relevant biological processes. GO functions upregulated in GO analysis were associated with chondrogenic differentiation, including regulation of nucleic acid-templated transcription, regulation of RNA biosynthetic process, transcription by RNA polymerase II, ECM organization, and anatomical structure morphogenesis. GO functions downregulated in GO analysis were associated with chondrogenic differentiation, including positive regulation of gene expression, positive regulation of nucleobase-containing compound metabolic process, embryo development, positive regulation of transcription, DNA-templated, and positive regulation of nucleic acid-templated transcription (Figure 2). Finally, 331 upregulated and 297 downregulated genes were obtained with statistical significance from the GO terms we confirmed (*p* < 0.01, FDR < 0.05).

**FIGURE 3:**
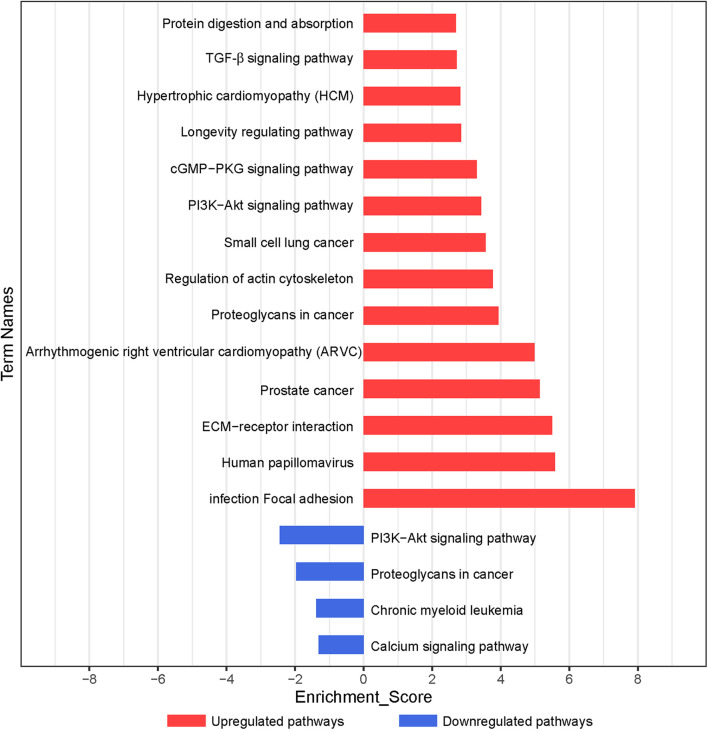
Pathway analysis of differentially expressed genes during chondrogenic differentiation. Significant KEGG pathways are represented on the *y*-axis. –LgP, negative logarithm of the *p*-value, are represented on the *x*-axis. Larger –LgP displays a smaller *p*-value.

According to the KEGG database, the significantly altered pathways involved in the functions of the differentially expressed genes were enriched. In total, 59 pathways, which may exert important effects on the chondrogenic differentiation of BMSCs (*p* < 0.05), were identified, with 55 upregulated and 4 downregulated pathways (Figure 3 and Supplementary Table 4). As shown in Figure 3, significant pathways enriched by upregulated genes included focal adhesion, ECM–receptor interaction, regulation of actin cytoskeleton, PI3K–Akt signaling pathway, cGMP–PKG signaling pathway, and transforming growth factor-β (TGF-β) signaling pathway. Significant pathways enriched by downregulated genes included the PI3K–Akt signaling pathway and calcium signaling pathway (Supplementary Table 4).

**FIGURE 4:**
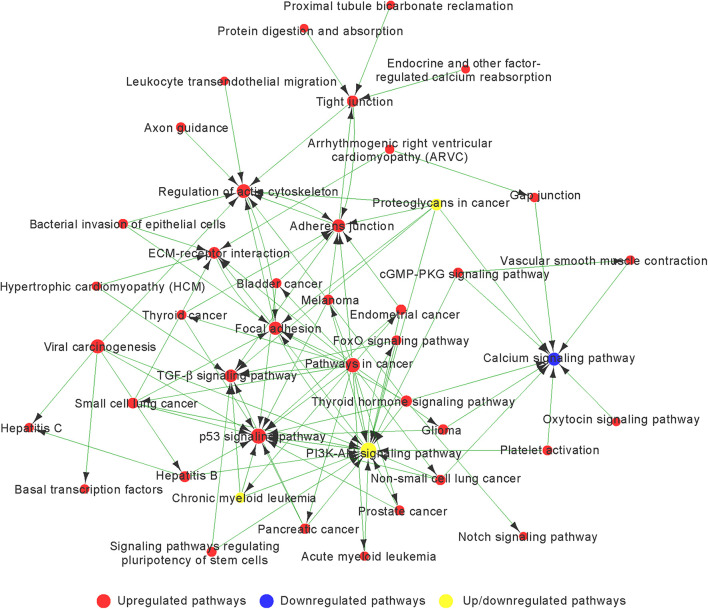
The interaction network of significant pathway (Path-Net) analysis of differentially expressed genes during chondrogenic differentiation. Nods represent different signaling pathways. Red and blue represent upregulated and downregulated pathways, respectively. Yellow represents both upregulated and downregulated pathways. Lines indicate a trigger relationship between significant pathways. The size of circle reflects the number of other pathways interacting with this pathway.

Based on the *p*-value, we determined 85 upregulated and 11 downregulated genes involved in significant pathways associated with chondrogenic differentiation (*p* < 0.05; Supplementary Table 5). We built Path-Net to identify the interactions among these significant pathways. The line between two nods indicates a trigger relationship between significant pathways. The degree of each pathway, which was represented by the size of circle, reflects the number of other pathways interacting with this pathway. Based on Path-Net, we found that crosstalk among these significant pathways may be critical in the chondrogenic differentiation of BMSCs (Figure 4). Our results identified the crosstalk among the PI3K–Akt signaling pathway, p53 signaling pathway, TGF-β signaling pathway, focal adhesion, and regulation of actin cytoskeleton signaling pathway.

We observed a highly increased activity of the p53 signaling pathway, focal adhesion, regulation of actin cytoskeleton, adherens junction, TGF-β signaling pathway, and ECM–receptor interaction associated with upregulated genes, and decreased activity of the calcium signaling pathway associated with downregulated genes (Figure 4 and Supplementary Table 6).

**FIGURE 5:**
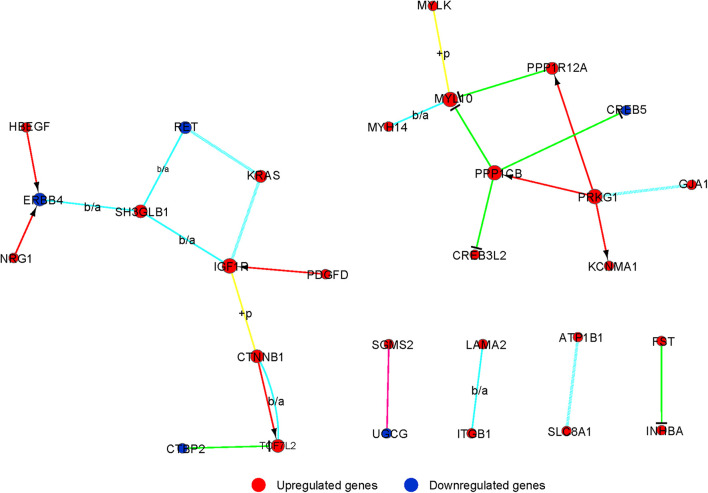
The interaction network of differently expressed gene (signal-net) analysis during chondrogenic differentiation. The color and shape of lines indicate interactions between genes.

Signal-net was built, an interaction network, to illustrate the interactions between differentially expressed mRNAs. Finally, we identified 29 mRNAs as potential core genes and regulatory relationships among these genes during the chondrogenic differentiation of BMSCs (Figure 5 and Supplementary Table 7).

**FIGURE 6:**
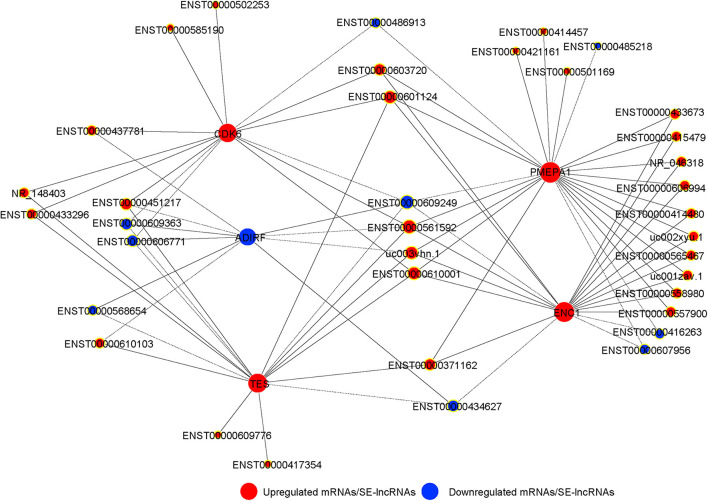
The co-expression network of SE-lncRNAs and mRNAs during chondrogenic differentiation of BMSCs. The nod with a yellow border represents lncRNA, and the nod without yellow border is mRNAs. Red represents upregulated SE-lncRNA/mRNA, and blue represents downregulated SE-lncRNA/mRNA, respectively. Connections of nods show the regulatory role between mRNAs and SE-lncRNAs. Solid lines indicate the positive correlation, and dashed lines indicate the negative correlation.

We integrated the differentially expressed SE-lncRNAs and mRNAs associated with these SE-lncRNAs. Five candidate genes (PMEPA1, ENC1, TES, CDK6, and ADIRF) were identified as possible core regulatory genes of BMSCs (fold change >3, *p* < 0.05; Supplementary Table 8). We calculated Pearson’s correlation coefficients of each pair of mRNAs (PMEPA1, ENC1, TES, CDK6, and ADIRF) and related SE-lncRNAs. Then, we constructed a CNC network for five mRNAs and SE-lncRNAs to confirm the interactions during chondrogenic differentiation of BMSCs according to the correlation coefficient. Solid or dashed lines connecting of nods indicate the regulatory role between mRNAs and SE-lncRNAs. As shown in Figure 6, several SE-lncRNAs may be closely related to a single mRNA. Thirty-seven SE-lncRNAs identified as core regulatory genes may play key roles during the chondrogenic differentiation of BMSCs based on the correlation coefficient (correlation coefficient >0.9, *p* < 0.05; Figure 6 and Supplementary Table 9).

## Discussion

Articular cartilage is a special tissue without blood vessels, nerves, and lymph, which leads to its poor self-repair ability (Duarte Campos et al., 2012; Wang et al., 2019; Lu et al., 2020). Articular cartilage injury is a common clinical condition, but treatment is still difficult because of the characteristics of the articular cartilage (Lu et al., 2020). BMSCs are the dominant seed cell source for tissue engineering (Zhang et al., 2018; Ma et al., 2019). They can be isolated and induced to differentiate into chondrocytes (Xu et al., 2020). The differentiation of BMSCs is controlled by numerous molecules and signaling pathways, including lncRNAs (Zhang et al., 2017). Many previous studies have reported that SE-lncRNAs, a class of lncRNAs transcribed from SE regions, are associated not only with cancer or complex diseases (Xie et al., 2018; Peng et al., 2019) but also with cell differentiation and development as well (Ounzain et al., 2015; Alvarez-Dominguez et al., 2017). However, the function of SE-lncRNAs during chondrogenic differentiation of BMSCs remains ambiguous. Thus, it is critical for BMSC therapy to understand the regulatory mechanisms of SE-lncRNAs and associated mRNAs in the chondrogenic differentiation of BMSCs.

Here, we performed studies to elucidate the expression profile of SE-lncRNAs and SE-lncRNA-associated mRNAs during the chondrogenic differentiation of BMSCs. According to microarray analysis, we identified 77 SE-lncRNAs that were differentially expressed, including 47 upregulated and 30 downregulated SE-lncRNAs. Additionally, the neighboring genes, which overlapped with SE-lncRNAs or located within 50 kb of the transcription start site, were considered to be target genes of SE-lncRNAs. In total, 308 mRNAs were identified, including 245 upregulated and 63 downregulated mRNAs. Our analysis showed dynamically expressed profiling of SE-lncRNAs and mRNAs during the chondrogenic differentiation of BMSCs. Some of the altered genes were found to be associated with chondrocyte differentiation and chondrogenesis. For example, EGR1, early growth response-1, induces the expression of OPN in chondrocytes via MEK–MAPK signaling. Inhibiting EGR1 expression impairs chondrocyte differentiation (Chen et al., 2015). RUNX1 is expressed in the differentiation phase of chondrocytes. When overexpressed, chondrocyte differentiation is strongly promoted (Tang C.Y. et al., 2020). The expression of ADAM12, a disintegrin and metalloproteinase 12, is increased during chondrogenic differentiation. The involvement of ADAM12 in TGF-β/BMP signaling crosstalk can influence chondrocyte differentiation (Horita et al., 2019). In our results, the expression of EGR1, RUNX1, and ADAM12 was upregulated and the expression of SFRP1, IGFBP6, and ADRB2 was downregulated, which is consistent with previous research. This suggests that these SE-lncRNAs and mRNAs with significantly altered expression in our study may be the key factors that regulate chondrogenic differentiation of BMSCs. GO enrichment analysis revealed that the biological processes that differentially expressed target genes were mainly enriched, including regulation of nucleic acid-templated transcription, regulation of RNA biosynthetic process, transcription by RNA polymerase II, ECM organization, and anatomical structure morphogenesis. These GO functions may be critical in the chondrogenic differentiation of BMSCs. The chondrogenic differentiation of BMSCs is controlled by several signaling pathways. KEGG analysis showed that upregulated target genes were mostly involved in focal adhesion, ECM–receptor interaction, regulation of actin cytoskeleton, cGMP–PKG signaling pathway, TGF-β signaling pathway, and downregulated target genes in the calcium signaling pathway. ECM and its receptor are associated with chondrogenic effects on MSCs (Li et al., 2018; Yang et al., 2018; Luo et al., 2020; Wang et al., 2020). ECM promotes the proliferation of MSCs and the maturity of chondrogenic differentiation (Li et al., 2018). ITGB1, one of the factors in the focal adhesion signaling pathway, can activate the ERK signaling pathway to improve the chondrogenic differentiation of MSCs (Luo et al., 2020). As a member of the TGF-β superfamily, BMP2 can induce chondrogenesis by controlling downstream molecules, such as RUNX2 and SOX9 (Zhou et al., 2016). Moreover, a Path-Net was constructed to show the interactions among these significantly altered pathways. A high degree of crosstalk has been shown among the PI3K-Akt signaling pathway, p53 signaling pathway, TGF-β signaling pathway, focal adhesion, and the regulation of actin cytoskeleton. The PI3K–Akt signaling pathway plays crucial regulatory roles in the regulation of cell differentiation (Zang et al., 2019). It is highly correlated with RUNX2-dependent chondrocyte differentiation (Fujita et al., 2004). The alteration of germline stem cells in p53 mutants may indicate that p53 is essential for stem cell proper differentiation (Park et al., 2019). TAGLN, a TGF-β inducible gene, plays an important role in stem cell differentiation. These results suggest that these signaling pathways may be the key pathways controlling the chondrogenic differentiation of BMSCs by the formation of a signaling pathway network. Research has shown that signaling can be integrated into signaling networks independently or by forming signaling crosstalk networks that influence the activity of signaling molecules and induce specific activation and differentiation pathways (Hitchcock et al., 2008; Ivashkiv, 2009). Then, we integrated SE-lncRNAs and mRNAs differentially expressed between non-induced and 7-day induced BMSCs. We then screened out mRNAs associated with SE-lncRNAs, which are vital for inferring the functions of SE-lncRNAs. PMEPA1, ENC1, TES, CDK6, and ADIRF were identified as the core regulatory genes. PMEPA1 is a transmembrane protein and a target of TGF-β signaling. An investigation demonstrated that PMEPA1, which is a key modulator of bone resorption (Xu et al., 2019), can be powerfully induced by RANK–p38 MAPK pathway signaling and can upregulate RANKL expression (Funakubo et al., 2018). ENC1, a gene encoding kelch-related actin-binding proteins, functions in oxidative stress associated with cellular senescence by inhibiting translation of Nrf2 protein (Wang and Zhang, 2009). At the same time, Nrf2 was shown to be able to determine the self-renewal capability and potential differentiation of MSCs from humans (Yoon et al., 2016). In addition, ENC1 implicates cytoskeletal reorganization and changing the cell shape (Zhao et al., 2000). Researchers have found that TES, a focal adhesion protein, interacts with several focal adhesion or cytoskeletal proteins such as actin and VASP and affects cell migration and adhesion (Coutts et al., 2003; Schulte et al., 2016). TES is also a negative regulator of cell proliferation (Ali et al., 2020). Previous studies have shown that cells upregulate cyclin D to activate CDK4 and CDK6 and enter the cell cycle (Liu et al., 2020). The promotion of proliferation can be achieved with the STAT1-CCND1/CDK6 axis by accelerating the transition from G0/G1 to S phase (Yu et al., 2020). ADIRF is also known as C10orf116 in 3T3-L1 cells and is highly expressed in adipose tissue. Overexpression of ADIRF has been shown to increase the transcription of C/EBPα and PPARγ to facilitate adipogenic differentiation. Overexpression of ADIRF also promotes cell proliferation and inhibits apoptosis (Chen et al., 2013). Most of these mRNAs we screened out are involved in cytoskeletal changes, which have been proven to be closely linked to MSC differentiation (Viale-Bouroncle et al., 2012). In addition, they are also associated with other physiological processes, including cell proliferation, migration, and cell cycle. These results suggest that the five genes may be the key modulator of chondrogenic differentiation of BMSCs.

The CNC network shows the co-expression of SE-lncRNAs and mRNAs and implies core regulatory factors. In addition to the five core genes, 37 SE-lncRNAs were screened and identified as possible regulators of chondrogenic differentiation of BMSCs. For example, researchers have confirmed ENST00000437781, also called DUXAP8, epigenetically silences PLEKHO1 to promote cell proliferation and migration (Ma et al., 2017). The interaction between DUXAP8 and EZH2 has been found by RNA pull-down assays (He et al., 2020). Meanwhile, researchers have found that EZH2 can mediate H3K27me3 to promote ATOH8 methylation and suppress ATOH8 expression, leading to the inhibition of chondrogenic differentiation of MSCs (Liu et al., 2021). Therefore, SE-lncRNAs may participate in the chondrogenesis of BMSCs by regulating their transcription. In summary, our findings imply that SE-lncRNAs may influence the chondrogenic differentiation of BMSCs through the regulation of potential downstream target genes, such as PMEPA1, ENC1, TES, CDK6, and ADIRF. Regrettably, we did not conduct experiments to validate the molecular mechanisms and functions of these SE-lncRNAs and mRNAs screened out in the chondrogenic differentiation of BMSCs. Hence, further studies are required.

## Conclusion

We clarified the complete expression of SE-lncRNAs and mRNAs during the chondrogenic differentiation of BMSCs. Bioinformatic analyses revealed that the SE-lncRNAs and mRNAs are highly related to chondrogenic differentiation of BMSCs and potential regulatory mechanisms. We further predicted the possible core regulators through co-expression networks. Our study provides new insights for further research on MSC biology.

## Data Availability Statement

The datasets presented in this study can be found in online repositories. The names of the repository/repositories and accession number(s) can be found below: https://www.ncbi.nlm.nih.gov/geo/, GSE171106.

## Author Contributions

YJ conducted the experiments, analyzed the data, and wrote the manuscript. CZ and LL participated in data analysis. LG and JG prepared the figures. ZF participated in the study design. GY reviewed and edited the manuscript. All authors read and approved the final manuscript.

## Conflict of Interest

The authors declare that the research was conducted in the absence of any commercial or financial relationships that could be construed as a potential conflict of interest.

## Publisher’s Note

All claims expressed in this article are solely those of the authors and do not necessarily represent those of their affiliated organizations, or those of the publisher, the editors and the reviewers. Any product that may be evaluated in this article, or claim that may be made by its manufacturer, is not guaranteed or endorsed by the publisher.

## Abbreviations

BMSCs: bone marrow-derived mesenchymal stem cells
SE-lncRNAs: super enhancer long non-coding RNAs
ECM: extracellular matrix
MSCM: mesenchymal stem cell medium
Path-Net: the interaction net of significant pathways
Signal-Net: gene signal transduction networks
CNC: coding-non-coding co-expression
FDR: false discovery rate
RT-PCR: real-time polymerase chain reaction
GO: Gene Ontology
TGF- β: transforming growth factor- β.
